# Validation of the Caen Chronotype Questionnaire: Exploring the added value of amplitude and correlations with actigraphy

**DOI:** 10.1080/07420528.2025.2471887

**Published:** 2025-03-05

**Authors:** Robert Hickman, Daniel Lai Jie, Sukhi Shergill, Sylvain Laborde, Teresa C. D’Oliveira

**Affiliations:** a Institute of Psychiatry,Psychology & Neuroscience, King’s College London, London, UK; b National Institute for Health and Care Research, Maudsley Biomedical Research Centre, South London and Maudsley National Health Service Foundation Trust, London, UK; c School of Psychology and Life Sciences, Canterbury Christ Church University, Canterbury, UK; d National Institute for Health and Care Research, Applied Research Collaboration Kent, Surrey and Sussex, Canterbury,UK; e Kent and Medway Medical School, University of Kent and Canterbury Christ Church University, Canterbury, UK; f Institute of Psychology, German Sport University, Cologne, Germany

**Keywords:** Chronotype, sleep, circadian rhythm, morningness-eveningness, amplitude, questionnaire validation, confirmatory factor analysis, shift work

## Abstract

Chronotype self-report instruments are time and cost-efficient measures to profile diurnal or time-of-day preferences. The Caen Chronotype Questionnaire (CCQ) captures morningness and eveningness (CCQ-ME) and a circadian amplitude dimension for diurnal variation (distinctiveness; CCQ-DI). This study extends prior multilanguage validations for the English version of the CCQ. In total, 628 participants enrolled from a UK working population (mean age 30.34 ± 8.36 years, 61.3% female) including a subset of shift workers (*n* = 179; mean age 27.62 ± 5.95 years, 49.2% female). A subsample of participants also wore a consumer-grade actigraph device (Fitbit Charge 4) for seven days to compare chronotype estimates with objective sleep-wake parameters (*n* = 22; mean age 27.05 ± 3.99 years, 81.8% female, 90.9% worked standard daytime schedules, and 9.1% worked rotating shifts). All participants completed online chronotype measures, including the CCQ and Morningness-Eveningness Questionnaire (MEQ), depressive symptoms (Patient Health Questionnaire; PHQ-9), sleep quality (Pittsburgh Sleep Quality Index; PSQI), and other outcome measures. Results from the Confirmatory Factor Analysis (CFA) offer support for a two-factor structure of the CCQ in an English-speaking sample, highlighting how individual preferences for the timing of activities is associated with chronotype (morningness-eveningness; ME) and a second subjective amplitude dimension (DI). However, in contrast with the original CCQ structure, a more parsimonious solution and best overall fit involved the reduction of the original 16-item questionnaire (8 items per factor) to 4 ME items and 5 DI items. Convergent validity with the reduced CCQ scale (rME) and the MEQ was also established. The CCQ was sensitive in discriminating differences in actigraphic sleep-wake timings between morning-and evening-oriented individuals. Regression models demonstrated that amplitude (CCQ-DI) was a significant predictor explaining most of the variance in depressive symptoms (PHQ-9) compared to other variables. Overall, the English version of the CCQ was shown to be a robust tool in estimating chronotype in a sample of adults based in the UK.

## Introduction

Chronotype refers to an individual’s preference in performing activities at certain times including sleep-wake rhythmicity, also known as morningness (morning “lark”) or eveningness (evening “night owl”) types (Jones et al. 2019; Melo et al. 2017; Thun et al. 2012; Walsh et al. 2022). Across the population, there is a wide distribution of chronotypes (Roenneberg et al. 2022) but most fall somewhere in between the “extreme” early to late chronotype categories (Adan et al. 2012). Chronotype can be influenced, however, by inter-individual variations such as age, sex, genotype, and intrinsic behavioural differences (Chauhan et al. 2023; Fischer and Hilditch 2022; Klerman et al. 2022). Environmental or external (zeitgeber) signals also entrain the circadian system (Foster 2020) and differentially impact chronotype, particularly light exposure (light timing, intensity, or consumption behaviour) and societal or social factors (Porcheret et al. 2018; Skeldon et al. 2017; Sletten et al. 2023).

Circadian preference at the physiological level can be estimated through biomarkers such as core body temperature, phase entrainment from dim light melatonin onset (DLMO), salivary cortisol, or actigraphy (Roenneberg et al. 2019). Although these markers are precise, they are also expensive, time consuming, and burdensome to administer. Self-reported chronotype measures, meanwhile, are a cost-effective and non-invasive method with strong reliability. Validated chronotype instruments commonly used include the Morningness-Eveningness Questionnaire (MEQ; Horne and Ostberg (1976)), Munich ChronoType Questionnaire (MCTQ; Roenneberg et al. (2003)), and Composite Scale of Morningness (CSM; Smith et al. (1989)) (Adan et al. 2012; Evans and Hasler 2022). Self-report measures are typically unidimensional, however, and capture only morningness and eveningness preference. Additional parameters such as amplitude or period are not measured which can also distinguish circadian preference (Di Milia et al. 2013; Marcoen et al. 2015). The Caen Chronotype Questionnaire (CCQ; Dosseville et al. (2013)), building on the work of Oginska (2011), was developed to capture this additional subjective amplitude through a distinctiveness scale (DI). Circadian amplitude relates to the strength, magnitude, and range of diurnal fluctuation or individual functioning that varies across the day (Díaz-Morales et al. 2017; Dosseville et al. 2013; Oginska 2011; Randler et al. 2016). Previous multilanguage studies have validated the CCQ and amplitude dimension in Arabic, Dutch, German, Italian, Portuguese, Spanish, and French (Laborde et al. 2018). An English version of the CCQ, however, has not undergone a full validation procedure and was the main aim of this study. Moreover, no previous studies have objectively validated the CCQ measure against actigraphic sleep-wake data, and only a small number have explored both morningness-eveningness and amplitude dimensions alongside actigraphy (Faßl et al. 2019; Paciello et al. 2022).

Secondary analyses (in addition to the main validation procedure) explored correlates of CCQ chronotype dimensions with depressive symptoms and shift work. Converging evidence shows eveningness orientation (preference for later bed and wake times) across both adolescents and adults is associated with increased prevalence of sleep disturbances, risk of negative emotionality, depressive states, and mood or psychotic disorders (Au and Reece 2017; Bauducco et al. 2020; Crouse et al. 2021; Delorme et al. 2020; Haraden et al. 2017; Melo et al. 2017; Merikanto et al. 2013; Simor et al. 2015). Chronotype-mood disorder associations, for example, were found in a meta-analysis for 35 of 36 studies with degree of eveningness positively correlated to mood disorder symptom severity (Au and Reece 2017). Sleep inertia, the transitional state experienced from sleep to wakefulness, has more recently been implicated as a biomarker driving elevated risk and incidence of psychiatric disorders among evening chronotypes (Burns et al. 2024; Hilditch and McHill 2019). Greater circadian amplitude is also linked with elevated risk of affective disorders (Laborde et al. 2018; Nowakowska-Domagala et al. 2016), worse health outcomes (Díaz-Morales et al. 2017; Saksvik et al. 2011), and depressive symptoms (Oginska et al. 2017). Chronotype-mood links for more evening (“night owl”) types may result from desynchrony to modern societal patterns. Individuals who stay awake later and rise later, for example, have higher misalignment between their biological and social clock, more curtailed sleep, and social jetlag (Roenneberg 2023; Wittmann et al. 2006). Social clocks are typically oriented towards morning chronotypes with societal pressures to wake and rise early (e.g. for school or work), which is often incongruent for individuals with later evening chronotype preference (Gorgol et al. 2022; Roenneberg et al. 2019).

Shift workers (around 20% of the working population) are vulnerable to circadian misalignment as a result of desynchrony to natural sleep-wake rhythms and light-dark cycles (Hittle and Gillespie 2018; Wickwire et al. 2017). Chronotype and diurnal preference has been shown to modulate toleration of shift work and sleep-related impairments (Ahn et al. 2024; Booker et al. 2018; Juda et al. 2013; Wickwire et al. 2017). Morning types, for example, have poorer sleep and adjustment to night shift work in contrast to evening types. Eveningness, meanwhile, is associated with greater adjustment difficulties and sleep impairments to early morning shifts (Kervezee et al. 2021; Saksvik et al. 2011). Chronotype-adjusted shift schedules, meanwhile, that are tailored according to individual, personal diurnal preference, improve sleep (duration and quality), well-being, and reduce social jetlag among shift workers (Potter and Wood 2020; Vetter et al. 2015).

To date, no prior work has fully validated the English translation of the CCQ. Hence, this study aimed to address four main research objectives: (1) First, to establish the construct validity and two-factor structure of the CCQ in an English-speaking population; (2) Second, to validate chronotype estimates (CCQ and MEQ) against objective consumer-grade actigraphic parameters (sleep onset and sleep-wake timings across workdays and free days); (3) Third, to perform secondary analyses of the added benefit and incremental validity of the amplitude dimension (CCQ-DI) which are hypothesised to predict depressive symptoms and sleep quality in line with prior studies (e.g. Oginska et al. (2017)); (4) Fourth, to assess circadian flexibility amongst shift workers, a group hypothesised to have lower amplitude (distinctiveness scores). The first and second objectives thus formed part of the main validation procedure and the third and fourth aims were secondary analyses.

## Study 1

### Material and Methods

#### Participants and Procedure

A total of 1,459 participants across the UK enrolled in the study with 831 excluded due to missing data from the MEQ, CCQ, or PHQ-9 measures. Sample characteristics for the final participants (*n* = 628) included in the analyses are outlined in Table 1. Final sample size for Study 1 is based on prior recommendations for instrument validation and comparable studies (Anthoine et al. 2014; Bacchetti 2010; White 2022). Participants were aged between 18 and 64 (*M* age 30.34 ± 8.36 years; 61.3% female) with a subsample of shift workers (*n* = 179; *M* age 27.62 ± 5.95 years, 49.2% female) who had fixed (*n* = 62), rotating (*n* = 116), or alternate (*n* = 1) shift patterns.

**Table 1.** Demographic characteristics for the overall sample (*N* = 628), shift worker (*n* = 179) and fitbit (*n* = 22) subsamples.

	All Participants (*n* = 628)			Shift Worker Sample (*n* = 179)			Fitbit Sample (*n* = 22)	
	N (%)			N (%)			N (%)	
**Region of work**								
Scotland	62 (9.9%)			31 (17.3%)			–	
South East	73 (11.6%)			11 (6.1%)			5 (22.7%)	
East of England	79 (12.6%)			29 (16.2%)			–	
Greater London	242 (38.5%)			36 (20.1%)			17 (77.3%)	
Northern Ireland	25 (4.0%)			13 (7.3%)			–	
Wales	25 (4.0%)			7 (3.9%)			–	
North East	12 (1.9%)			6 (3.4%)			–	
North West	22 (3.5%)			13 (7.3%)			–	
Yorkshire and the Humber	19 (3.0%)			10 (5.6%)			–	
West Midlands	21 (3.3%)			9 (5.0%)			–	
East Midlands	11 (1.8%)			2 (1.1%)			–	
South West	36 (5.7%)			11 (6.1%)			–	
Missing	1 (0.2%)			1 (0.6%)			–	
**Sex**								
Female	385 (61.3%)			88 (49.2%)			18 (81.8%)	
Male	237 (37.7%)			88 (49.2%)			4 (18.2%)	
Non-Binary	2 (0.3%)			1 (0.55%)			–	
Did not specify	4 (0.7%)			2 (1.1%)			–	
**Age range**								
18–29	380 (60.5%)			130 (72.6%)			19 (86.3%)	
30–39	160 (25.5%)			37 (20.7%)			3 (13.7%)	
40–49	56 (8.9%)			8 (4.5%)			–	
50–59	21 (3.3%)			2 (1.1%)			–	
60–64	6 (1.0%)			–			–	
Missing	5 (0.8%)			2 (1.1%)			–	
**Main language**								
English	608 (96.8%)			175 (97.8%)			22 (100%)	
Other	15 (2.4%)			2 (1.1%)			–	
Missing	5 (0.8%)			2 (1.1%)			–	
**Type of work contract**								
Fixed	309 (49.2%)			93 (52%)			13 (59.1%)	
Permanent	315 (50.2%)			84 (46.9%)			9 (40.9%)	
Missing	4 (0.6%)			2 (1.1%)			–	
**Type of shift work**								
Non-shift work	449 (71.5%)			–			20 (90.9%)	
Rotating	116 (18.5%)			116 (64.8%)			2 (9.1%)	
Fixed	62 (9.9%)			62 (34.6%)			–	
Other	1 (0.1%)			1 (0.55%)			–	

Data collection occurred between 2021 and 2023. Recruitment was undertaken through a combination of email circulars, online webpages and posts, and social media channels. Informed consent was obtained and all measures administered online through Qualtrics. Individuals aged 18–65 years, working full-time, currently living in the UK, and who had English as their main speaking language were eligible. Participants were able to enter a raffle for a monetary incentive (voucher). Study protocol and ethical approval was obtained by the Research Ethics Committee at King’s College London (KCL).

#### Measures

##### The Caen Chronotype Questionnaire (CCQ)

The CCQ (Dosseville et al. 2013) consists of 16 items capturing Morningness-Eveningness (CCQ-ME; 8 items) and Distinctiveness (amplitude) dimensions (CCQ-DI; 8 items). The final version of the CCQ can be seen in Supplementary Table S1 and includes coding instructions (reversed items for 4 ME and 5 DI). Items in the CCQ-ME (e.g. “I feel I can think the best in the morning”) and CCQ-DI subscales (e.g. “I can work efficiently at any time of the day”) are rated on a Likert scale from 1 (*totally disagree*) to 5 (*totally agree*). Unlike other chronotype instruments, for which high scores denote higher degrees of morningness, a high score on the CCQ-ME scale indicates higher eveningness (Dosseville et al. 2013; Laborde et al. 2018; Randler et al. 2015). Meanwhile, a high score on the CCQ-DI scale denotes a higher subjective amplitude.

##### The Morningness-Eveningness Questionnaire (MEQ)

The MEQ (Horne and Ostberg 1976) is a 19-item measure of morningness and eveningness (ME), with similarities to the CCQ-ME subscale. Large scale studies have demonstrated its strong validity and reliability and is considered the gold standard in capturing subjective chronotype estimates (Levandovski et al. 2013). Certain items include time scales (e.g. “At what time would you get up if you were entirely free to plan your day?”). MEQ scores range from 16 to 86 with evening type (≤41), intermediate type (42–58), or morning (≥59) type. Low scores (16–30) denote “*Definitely evening type*” and high scores (70–86) denote “*Definitely morning type*.”

##### Patient Health Questionnaire (PHQ-9)

The PHQ-9 (Kroenke and Spitzer 2002) is a validated depressive symptom screening tool, often used in primary and secondary care settings (Shevlin et al. 2022). Nine questions are scored (0–3) with a maximum summed score of 27. Threshold scores for mild depression (≥10), moderate depression (≥15), and major depression (≥20) are commonly used, whilst a prior systematic review concluded that cut-off scores between 8 and 11 are acceptable in detecting major depressive disorder (Manea et al. 2012).

##### Pittsburgh Sleep Quality Index (PSQI)

The PSQI (Buysse et al. 1989) is a 24-item measure of seven sleep parameters: sleep quality (SQ), sleep latency (SOL), sleep duration (TST), habitual sleep efficiency (SE), sleep disturbances, use of sleep medication, and daytime function. Items are summed to generate a *global PSQI* score (0–21) with higher scores indicating poorer sleep quality over the past month. A cut-off *global PSQI* score of >5 is indicative of poor sleep quality.

##### Additional Measures and Lifestyle Factors

Sociodemographic questions, job information, work and shift patterns, lifestyle (e.g. caffeine consumption, tendency to nap, physical activity) were also completed in line with prior CCQ validation studies (Laborde et al. 2018). Additional measures (not reported or analysed here) included the Global Sleep Assessment Questionnaire (GSAQ) (Roth et al. 2002), Karolinska Sleepiness Scale (KSS) (Akerstedt and Gillberg 1990; Akerstedt et al. 2014), General Practice Physical Activity Questionnaire (GPPAQ) (Smith et al. 2017) and Work Intensity (5-item) measure (Brown and Leigh 1996).

## Study 2 – Actigraphy

Recruitment procedures were identical to that of Study 1 and ethical approval was obtained by the Research Ethics Committee at King’s College London (KCL). After completing the online self-report measures from Study 1, a subsample of participants (*n* = 22) wore a consumer-grade wearable device on their non-dominant wrist for seven continuous days. The device (Fitbit Charge 4) recorded daily sleep-wake and rest-activity patterns. A recent systematic review found that, compared to polysomnography (PSG), the Fitbit Charge 4 is relatively accurate and sensitive in estimating sleep stages or parameters (Schyvens et al. 2024). In this study, actigraphic sleep indices of interest were sleep onset and sleep-wake timing across workdays and free days. Firmware versions were consistent across devices in the study. Minor updates during data collection meant that 18 devices had firmware version (48.20001.78.33), 3 devices had version (48.20001.100.76), and 3 devices had version (48.20001.100.43).

Participants from the Study 2 subsample were aged between 18 and 39 years old (*M* age = 27.05 ± 3.99 years, 81.8% female). All participants were employed, with 20 individuals (90.9%) working standard daytime schedules and 2 individuals (9.1%) working rotating shift patterns. Sample characteristics are outlined in Table 1. The sample size for Study 2, however, was limited due to pragmatic considerations such as study timeframe, available resources, and equipment.

## Data Analysis

### Confirmatory Factor Analysis (CFA)

Data^1^ was checked for normality and assumptions of a Confirmatory Factor Analysis (CFA) were based on recommendations outlined by Harrington (2008). All items were normally distributed (kurtosis ranged from −1.19 to 0.87; skewness ranged from −0.47 to 0.35). A Kaiser-Meyer-Olkin Measure of Sampling Adequacy (KMO) test (value of 0.829) and Bartlett’s test of Sphericity (*p* < 0.001) determined factor analysis eligibility. The 16 items of the CCQ were normally distributed and so a CFA was performed.

CFAs were run using maximum likelihood estimation with SPSS AMOS 29 to evaluate model adequacy and fit. When more than one latent factor was considered in the model, factors were allowed to correlate given that both refer to the same conceptual domain of Chronotype. This procedure allowed us to test distinct theoretical approaches empirically.

The CFA compared two main models and was designed based on the theory of past research: (1) One-factor model with all 16 CCQ items (Supplementary Figure S1). This model reflects the existing literature on Chronotypes that identifies one main latent factor with two extreme values, Morningness and Eveningness. (2) Two-factor model with 8 items on Morningness-Eveningness (CCQ-ME) and 8 items on Distinctiveness (CCQ-DI) (Supplementary Figure S2). This model is directly associated with the work on the CCQ that proposed a 2-factor solution, with 1 factor on Morningness and Eveningness and 1 factor on Distinctiveness. After running the initial analysis, factor loadings for items below 0.5 were removed, as per guidelines by Tabachnick et al. (2013) and modification indices were checked to improve model fit. These recommendations resulted in a reduced 2-factor model (R2) with 4 items on Morningness-Eveningness (CCQ-ME) and 5 items on Distinctiveness (CCQ-DI) (Supplementary Figure S3). In total, our results analysed and compared these three models.

The models were compared for how well the data fit, its convergent and discriminant validity, and reliability. For model fit, the fit indexes Chi-squared (χ^2^), Comparative Fit Index (CFI), Tucker-Lewis Index (TLI), and the Root Mean Square Error Approximation (RMSEA) were used. A “good” fitting model was based off prior criterion (Bentler 1992; Hu and Bentler 1999) with large chi-squared values (χ^2^) indicating a “poor” fit and small values a “good” fit. Chi-squared values, however, are sensitive to sample sizes with larger samples giving inflated values (Bentler 1990). CFI and TLI fit indices suggest acceptable fit for values (0.90–0.94) and relatively good fit for those above 0.95 whilst RMSEA values smaller than 0.08 indicate an acceptable fit and below 0.05 for a “good” fit (Hu and Bentler 1999).

### Convergent and Discriminant Validity

Convergent validity refers to the degree a construct is well measured by its indicators, whilst discriminant validity is the degree to which different constructs are not related. Within the literature, there are various accepted procedures of how convergent and discriminant validity are evaluated. The following procedures were used to ensure a robust analysis of convergent and discriminant validity amongst the CCQ scales:

1. The CCQ-ME should measure the same construct measured by the MEQ and the CCQ-DI should be a distinct construct from the MEQ. This was assessed using Pearson’s correlations tests between the MEQ and CCQ scales, as proposed by previous CCQ validation studies (e.g. Dosseville et al. (2013), Laborde et al. (2018)).
2. The internal structure and dimensionality of each CCQ construct was assessed using the Fornell and Larcker (1981) criterion, based on recommended practice by Cheung et al. (2024). The criterion uses the average amount of variance a construct explains through its indicators, in relation to the overall variance among all indicators. Based on the criterion (Fornell and Larcker 1981), convergent validity is established when the Average Variance Extracted (AVE) by a construct is ≥0.5 or when Composite Reliability of a construct is >0.6 when AVE is < 0.5. The discriminant validity between two factors is established when the square root of AVE is greater than the correlation between factors.

### Internal Consistency and Reliability

Cronbach’s alpha (α) and composite reliability (CR) were used to evaluate the CCQ internal consistency and reliability. CR and α values > 0.70 show adequate reliability (Bagozzi and Yi 1988; Cronbach 1951).

### Incremental Validity of Amplitude

Hierarchical multiple linear regressions were used to assess the incremental validity of amplitude (DI) in predicting depressive symptoms (PHQ-9). Data were entered in blocks with the first block including age and sex, the second block entering morningness-eveningness (ME) scale of the CCQ, and the third block included the DI dimension. For shift workers, the incremental validity of amplitude (DI) in predicting depressive symptoms (PHQ-9) and sleep quality (PSQI) was also assessed. Two models were constructed with depressive scores (PHQ-9) as the dependent variable in one model and sleep quality scores (PSQI) as the dependent variable in the other model.

## Results

### Confirmatory Factor Analysis (CFA)

The one-factor model had poor fit ($\chi^{2}$*=* 1913.45, d*f* = 104, CFI = .42, TLI = .34, RMSEA = .166). A two-factor model improved the fit but was also unacceptable ($\chi^{2}$*=* 1373.45, d*f* = 103, CFI = .59, TLI = .46, RMSEA = .109). Problematic items were identified via analysis of factor loadings to improve the model fit.

Factor loadings of the CCQ-ME factor were considered “very good” (>.63) for items I2, I11, and I13, “good” (>.55) for item I7, “fair” (>.45) for item I16, and “poor” (<.44) for items I4, I5, and I9. CCQ-DI factor loadings were “very good” (>.63) for items I8 and I10, “good” (>.55) for items I1 and I6, “fair” (>.45) for item I12 and “poor” (<.44) for items I3, I14, and I15 (Tabachnick et al. 2013). Factor loadings for items below 0.5 were removed (see Table 2) for CCQ-ME (I4, I5, I9, I16) and CCQ-DI (I3, I14, I15).

**Table 2.** Fit indices for the confirmatory factor analysis (CFA).

Model	Chi- Squared (χ^2^)	df	RMSEA	CFI	TLI
M1: Reduced Two-Factor (R2)	54.22	24	.045	.98	.97
M2: Two-Factor	1373.45	103	.109	.59	.46
M3: One-Factor	1913.45	104	.166	.42	.34
Null Model	3281.93	120	.205	–	–

Table presents the fit indexes of the three models tested. Chi-Squared (χ^2^) goodness of fit test; df = Degrees of freedom; RMSEA = Root Mean Square Error Approximation; CFI = Comparative Fit Index; TLI = Tucker-Lewis Index.

Model fit was refined by removing low loading factors and by following recommendations by Modification Indices (MI; refer to Supplementary Figure S4) when theoretically appropriate. The next MI (value = 29.48) suggested correlating error variance of Item 6 and Item 10. The last MI (value = 14.55) recommendation resulting in strong model improvement was the correlation between error variances of Item 2 and Item 13. After these revisions, the model fit was good ($\chi^{2}$ = 54.22, d*f* = 24, CFI = .98, TLI = .97, RMSEA = .045; refer to Table 2 for all fit indexes). Factor loadings on the ME factor of the Reduced two-factor (R2) model were all “very good” (>.63), and for the DI factor I1, and I8 are “very good,” I10 is “good” (>.55), and “fairly” (>.45) for I6, and I12 (both items = .53; refer to Table 3). The new scales are denoted as rME and rDI.

**Table 3.** Factor loadings of the Two-factor and reduced Two-factor (R2) model.

Item	Two-Factor Model		Reduced Two-Factor (R2) Model	
	ME	DI	ME	DI
1 2 3	.**77**	.**61** .20	.**65**	.**65**
4 5 6	.34 .40	.**59**		.**53**
7 8 9	.**58** .40	.**75**	.**67**	.**78**
10 11 12	.**68**	.**67** .**51**	.**76**	.**62** .**53**
13 14 15	.**70**	.24 .35	.**66**	
16	.49			

**Bolded** factor loadings were included into the Reduced 2 factor model (R2). Underlined factor loadings did not meet the >0.5 criteria. All factor loadings are significant (*p* < 0.05).

### Convergent and Discriminant Validity

As expected, significant and positive correlations between the initial and the revised dimensions of the CCQ scales were observed: *r* = .82, *p* < 0.001 for the ME scale and the new reduced ME (rME) scale and *r* = .85, *p* < 0.001 for the proposed DI scale and reduced DI (rDI) scale. Pearson’s correlation evaluated how the reduced CCQ scales converged with the gold standard MEQ. Pearson’s correlations showed a significant, negative, and medium association between rME and MEQ (*r* = −.52, *p* < 0.001) and a significant, negative, weak association between rDI and MEQ (*r* = −.20, *p* < 0.001).

The AVE for the one-factor model was 0.20. The AVE for the two-factor model was 0.32 for ME and 0.28 for DI. The AVE for the reduced model was 0.48 and 0.39 for rME and rDI respectively. Although the models under analysis did not meet the AVE ≥ 0.5 criteria, based on the Fornell and Larcker (1981) criterion, convergent validity of the reduced two-factor model was met, as the composite reliability of both factors is >0.6 (reported in the section below).

The square root AVE of ME and DI was greater than the correlation between the factors ($\sqrt{\mathrm{MEAVE}}$= .57; $\sqrt{\mathrm{DIAVE}}$= .53; *r* = .40, *p* < 0.001). The criterion was also met for the R2 model ($\sqrt{\mathrm{rMEAVE}}$ = 0.69; $\sqrt{\mathrm{rDIAVE}}$ = 0.63; *r* = .58, *p* < 0.001). Thus, good discriminant validity is established for the two-factor and reduced two-factor (R2) models. The data suggests that the two factors can be both theoretically and empirically distinguished.

### Internal Consistency and Reliability

Internal consistency was calculated using Cronbach’s alpha (α). As reported in Table 4, all three models presented alpha values (α) above 0.7. The one-factor model had acceptable internal consistency (α= .78). The two-factor model had acceptable internal consistency for the ME (α = .79) and DI (α= .73) scales. The R2 model produced the highest internal consistency for the rME and rDI compared to the two-factor and one-factor models. Good internal consistency was achieved by the rME (α= .80) and an acceptable internal consistency for the rDI (α= .77). Furthermore, results for Composite Reliability (CR) for all models also met the recommended cut-off levels (OneFactorCR = .76; MECR = .78, DICR = .73; rMECR = .79, rDICR = .76) with the R2 model demonstrating higher CR values than the two-factor model.

**Table 4.** AVE, Cronbach’s alpha, and composite reliability for all three models.

Model	AVE		α		CR	
One-Factor	.20		.78		.77	
	ME	DI	ME	DI	ME	DI
Two-Factor	.32	.28	.79	.73	.78	.73
Reduced Two-Factor (R2)	.48	.39	.80	.77	.79	.76

R2 = Reduced Two-Factor Model; AVE = Average Variance Extracted; = Cronbach’s Alpha; CR = Composite Reliability.

## Incremental Validity of Amplitude Predicting Depressive Symptoms

To assess the added value of including an assessment of circadian amplitude (DI scale) when predicting depressive symptoms (PHQ-9), a hierarchical multiple linear regression analysis was used. Age and sex were entered as a first block resulting in an insignificant model (*p* = 0.41). The $R^{2}$ value of 0.004 obtained from the regression model suggests that sex and age accounted for 0.04% of the variation of depressive symptom scores. ME was entered as a second block resulting in a statistically significant change (*p =* .016) of the regression model. The $\Delta R^{2}$ value of 0.009 suggest that ME scores accounted for 0.09% of the variation in depressive symptom scores. Finally, amplitude (DI) was added as a predictor in the third block, a regression model that was found to be statistically significant (*p =* .003). DI results accounted for 1.4% variation in depressive symptom scores $(\Delta R^{2}$ = 0.014). When controlling for Age, Sex, and ME, the regression coefficient [β = 0.13, 95% CI (0.043,0.209), *p* = 0.003] associated with DI suggests that with each additional unit of DI, PHQ-9 scores increased by approximately 0.13 points. A similar analysis was conducted entering DI in the second block and ME in the third block. In contrast with the previous analysis, when controlling for Age, Sex, and DI, the ME predictor variable is not statistically significant (*p* =0.096), a result that emphasises the unique contribution of DI to depressive symptoms.

Overall, when predicting depressive symptoms, the inclusion of amplitude (DI) as a predictor rendered the regression model significant and incrementally explains 2.1% of the variation of these affective experiences (refer to Table 5 for the change statistics of each predictor).

**Table 5.** Incremental validity of the DI scale in predicting depressive symptoms (PHQ-9).

				Change Statistics		
Predictor	$R^{2}$	SE	$\Delta R^{2}$	F	df	*p*
Sex + Age	.004	5.337	.004	1.324	(2, 618)	0.41
ME	.014	5.316	.009	5.852	(1, 617)	0.016*
DI	.021	5.282	.014	8.960	(1, 616)	0.003*

Table 5 presents the results of the hierarchical regression with depressive symptoms being the outcome variable. Change in $R^{2}$ values and the F-test p-values denote if a significant change was found when the predictor variable is added into the regression model. ME = Morningness and Eveningness; DI = Distinctiveness; *p < 0.05.

## Shift Workers

### Chronotype Estimates, Depressive Symptoms, and Sleep Quality

For the subsample of shift workers, relationships between chronotype estimates (CCQ-ME, CCQ-DI, and MEQ) and self-reported sleep quality and depressive symptoms were assessed.

#### CCQ

No significant linear relationships were found between depressive symptoms (PHQ-9) and CCQ-ME (*r* = .124, *p* = 0.099) or CCQ-DI (*r* = −.103, *p* = 0.170) dimensions. No significant linear relationships were also found between subjective sleep quality (PSQI) and CCQ-ME (*r* = .119, *p* = 0.113) or CCQ-DI (*r* = .034, *p* = 0.649).

#### MEQ

No significant linear relationships were found between MEQ scores and subjective sleep quality (PSQI; *r* = −.073, *p* = 0.339) or depressive symptoms (*r* = −.108, *p* = 0.153).

As no linear relationships were found amongst shift workers between chronotype estimates (CCQ or MEQ) and sleep quality or depressive symptoms, hierarchical regression modelling was not performed.

### Shift Worker Morning-Evening Types

Chronotype amplitude estimates (CCQ-DI) were also compared between shift and non-shift workers. When shift workers (*M* = 24.31, *SD* = 4.865) were compared with non-shift workers (*M* = 25.98, *SD* = 5.483), shift workers had significantly lower distinctiveness scores, t (366.64) = 3.74, *p* < 0.001 with a small effect size (d = .314). On average, this suggests shift workers have less rigid subjective amplitude and more flexibility in tolerating circadian variation.

## Study 2 – Actigraphy CCQ Validation

### Actigraphy Correlations

The relationships between the CCQ and MEQ chronotype measures with consumer-grade actigraphic sleep-wake parameters (Fitbit Charge 4) are shown in Table 6.

**Table 6.** Pearson and Spearman’s Rho correlations between chronotype measures (CCQ and MEQ) and consumer-grade actigraphic sleep parameters.

Actigraphic sleep parameter	CCQ-ME	CCQ-DI	MEQ
*Sleep onset – workday ^a^*	rho = .597*	rho = .340	rho = −.682**
*Sleep onset – free day*^*a*^	rho = .590*	rho = .323	rho = −.713**
Wake time – workday	r = .617*	r = .375	r = −.654**
Wake time – free day	r = .337	r = .128	r = −.670**
*Mid-point sleep – workday*^*a*^	rho = .424	rho = −.238	rho = −.346
*Mid-point sleep – free day*^*a*^	rho = .341	rho = −.101	rho = −.214
*Sleep efficiency (SE)*^*a*^	rho = −.164	rho = −.052	rho = .173

rho = Spearman’s Rho; **r** = Pearson Correlation; CCQ-ME = Morningness-Eveningness dimension; CCQ-DI = Distinctness dimension; *p < 0.05 and **p < 0.01, *Italicised variables* ^*a*^ were not normally distributed and so Spearman’s Rho correlations were reported.

#### CCQ-ME

The CCQ morningness-eveningness (CCQ-ME) dimension had significant moderate associations with actigraphic sleep onset (workdays and free days) and rise times (workdays). Positive correlations demonstrate individuals with higher CCQ-ME scores (denoting high degrees of eveningness) have later actigraphic recorded sleep onset timings on both workdays and free days as well as later wake times on workdays.

#### CCQ-DI

Correlation analyses for the amplitude dimension (CCQ-DI) showed no significant association with sleep-wake timings. This suggests the CCQ-DI scale may capture distinct dimensions from sleep-wake timing preference. Actigraphic data in this study did not capture intra-daily and inter-daily variability which may be more related to the amplitude dimension (CCQ-DI).

#### MEQ

The MEQ significantly correlated with actigraphic sleep-wake times, as expected from prior extant studies.

#### Actigraphy Morning-Evening Types

CCQ chronotype scores were assigned according to quartiles for Morning (≤18) and Evening type groups (≥27) and based on the distribution of the larger UK-wide sample in Study 1 (*N* = 628). This followed prior classification procedures (e.g. (Paciello et al. 2022; Thun et al. 2012), and consisted of 7 Morning types, 9 Evening types, and 6 Intermediate types for the Study 2 subsample. Overall, based on the Mann Whitney U Test comparing the Morning and Evening group types, the CCQ was a good tool in discriminating Morning and Evening types from objective actigraphic sleep-wake timings.

#### Sleep Onset

Sleep times were significantly different between morning and evening types on workdays, (U = 6.00, *p* = 0.007). Morning types had earlier sleep times (median = 23:13:53, SD = 0:37:37) compared to evening types (Median = 00:33:06, SD = 1:29:31). For free days, there was also a significant difference between sleep times for morning and evening types (U = 9.00, *p* = 0.022). Morning types also slept earlier (Median = 23:54:09, SD = 0:52:36) compared to evening types (Median = 01:40:02, SD = 1:20:21).

#### Wake Times

Rise times significantly differed between morning and evening types on workdays (U = 8.00, *p* = 0.016). Morning types had earlier wake times (median = 07:22:36, SD = 1:10:21) compared to evening types (Median = 09:03:43, SD = 1:03:24). No significant differences were observed between rise times on free days of morning and evening types (U = 17.00, *p* = 0.181). Whilst there were no significant differences, morning types had earlier median wake times (Median = 08:58:15, SD = 0:55:25) compared to evening types (Median = 09:45:15, SD = 0:44:35).

## Discussion

The main objective of this study was to validate the Caen Chronotype Questionnaire (CCQ) in an English-speaking sample (see Table S1 for the full CCQ version). It aimed to validate the CCQ factor structure proposed by Dosseville et al. (2013), evaluate the internal consistency, convergent and discriminant validity, and to correlate findings with consumer-grade actigraphic parameters. Secondary analyses explored the incremental predictive validity of the CCQ amplitude dimension (distinctiveness; CCQ-DI scale) on depressive symptoms and sleep quality. Lastly, chronotype estimates and circadian flexibility of shift workers was assessed.

### Key Findings

1. The best fitting model of the English-translated CCQ consisted of a two-factor structure with distinguishable ME and DI parameters. These findings are in line with prior multilanguage validation studies from Laborde et al. (2018) in an English language sample.
2. Although a two-factor structure was confirmed, the original two-factor structure of the English-translated CCQ had unacceptable fit. A good fitting reduced two-factor version of the CCQ was proposed.
3. A reduced CCQ measure capturing diurnal preferences was also psychometrically sound and the most parsimonious.
4. The CCQ discriminated morning and evening types from consumer-grade actigraphic parameters. Further validation with sufficient sample size and research/clinical-grade actigraphy is needed.
5. Circadian amplitude incrementally predicted depressive symptoms. Future research should further assess depressive subgroups.
6. Shift workers had less rigid subjective amplitude (diurnal fluctuation) compared to day workers, which may be advantageous in tolerating shift-work-related circadian misalignment.

### Two-Factor CCQ Model

Compared with the competing one-factor model, factor analysis suggests a two-factor CCQ structure is best. Fit indices of the original 16-item CCQ, however, did not fit the data well. Using factor loadings, residuals and modification indices, the two-factor model was modified into a revised model. The reduced two-factor (R2) model had 4 items loaded onto the ME factor and 5 items loaded on the DI factor. The R2 model had the best fit indices values when compared to the other two models. Moreover, it was the only model that met the criteria of a “good fitting” model outlined in Hu and Bentler (1999). The R2 model also produced the highest Cronbach Alpha (α) and Composite Reliability (CR). Although the reliability of the amplitude (DI scale) has been an issue for previous CCQ validation studies (Dosseville et al. 2013; Laborde et al. 2018; Oginska 2011), the DI scale in our study had acceptable internal consistency and composite reliability.

### Convergent Validity

Convergent validity for the reduced ME scale (rME) was generally acceptable based on Average Variance Extracted (AVE =.48; CR = .79) (Fornell and Larcker 1981). Correlations were also found between the reduced morningness-eveningness dimension of the CCQ (rME) and MEQ measure (Horne and Ostberg 1976), further establishing convergent validity. Moderate negative correlations were expected, since higher CCQ-ME scores denote greater degrees of eveningness whilst higher MEQ scores denote greater morningness. Low correlations between the amplitude (DI scale) and MEQ (*r* = −.20) measure demonstrate that the subjective amplitude dimension captures a chronotype estimate which is distinct from morningness-eveningness preference. Like the CCQ-ME scale, the AVE of the DI scale was <.5, but was deemed adequate (high composite reliability >.6).

### Discriminant Validity

Discriminant validity of the reduced amplitude scale (rDI) was assessed with correlations with the MEQ which support prior findings from Dosseville et al. (2013). The observed weak correlation between the variables suggests that the rDI scale is addressing a concept distinct from Morningness and Eveningness.

### Incremental Validity of Amplitude

Amplitude (CCQ-DI), but not morning-eveningness (CCQ-ME), increased the predictive ability of reported depressive symptoms, although the variance was small. Prior research suggests eveningness is associated with depressive symptomology (Bauducco et al. 2020), but when amplitude was controlled for in our study, the ME scale did not significantly predict depressive scores. Discrepancies in findings may, in part, be explained by the use of single, unidimensional scales in previous studies and larger or more representative samples (e.g. Merikanto et al. (2015)). Future research should consider the potential moderating effect of amplitude (CCQ-DI) when discussing sleep consistency. Sleep consistency, also described as “sleep regularity,” refers to a person’s sleep-wake timing stability. In other words, how constant or regular their sleep schedule is over multiple nights. This sleep regularity dimension has recently been recognised as a predictor of health outcomes, including depressed mood and mortality risk (Sletten et al. 2023; Windred et al. 2024; Zuraikat et al. 2023). Based on our results, there is a need to understand if sleep consistency is a strong predictor across all profiles of amplitude or mainly for those with more rigid circadian alignment.

### Shift Workers

Amongst shift workers, the incremental validity of amplitude (CCQ-DI) in predicting depressive symptoms was also evaluated. No significant correlations were found for CCQ dimensions and sleep quality or depressive symptoms, and so regression modelling was not performed. Shift workers, however, were shown to have significantly lower amplitude (DI scores) which may indicate greater flexibility in tolerating circadian misalignment compared to day workers. Future research should capture more granular shift type details such as scheduling patterns and frequency of shift rotation. Diverse shift schedules with larger sample sizes are needed to elucidate these associations.

### Actigraphy

Individuals with higher CCQ-ME scores (high degrees of eveningness) had later actigraphic recorded sleep onset timings (workdays and free days) and later wake times (workdays). No associations were found between amplitude scores and actigraphic parameters (sleep onset and rise times), suggesting the CCQ-DI scale captures distinct dimensions (i.e. diurnal fluctuation) from sleep-wake timing preference. Overall, the CCQ was a robust tool in discriminating Morning and Evening types from objective consumer-grade actigraphic sleep-wake timings. Morning types (based on CCQ quartiles) went to bed earlier compared to evening types for both work and free days. Morning types also rose earlier on workdays and had earlier median wake times compared to more evening types. Future studies should also develop standard CCQ cutoff scores, in addition to quartiles, to assess relationships with actigraphic parameters. The fit of the reduced two-factor CCQ (R2) model should also be considered.

### Limitations

A cross-sectional design was used in this study and so causality and direction of associations are not fully established. Prospective daily sleep markers (e.g. sleep diaries) together with additional physiological circadian parameters (e.g. core body temperature or melatonin) would strengthen findings. Self-report measures may have response bias and online data collection utilised in this study (whilst improving overall sample size) may have reduced response accuracy. The final sample did not have an equal sex distribution and female respondents were over-represented (61.3%), particularly in the Fitbit subgroup (81.8%). Most participants were also under 40 years of age. These two factors (sex and age) are known to effect chronotype: men, for example, typically have later (more evening-oriented) chronotypes (Randler and Engelke 2019), whilst women may have earlier circadian phase timings and faster circadian clocks (Lok et al. 2024; Vidafar et al. 2024). Sex differences in chronotype may even decrease or disappear in later adulthood, potentially reversing with age from around 40 to 50 years (Díaz-Morales and Parra-Robledo 2018; Fischer and Hilditch 2022; Randler and Engelke 2019; Roenneberg et al. 2004). The test-retest reliability of the CCQ English version, measurement invariance, and longitudinal stability also need further evaluation. Additional validation against other instruments such as the Munich Chronotype Questionnaire (MCTQ; (Roenneberg et al. 2003)) and the Morningness-Eveningness-Stability-Scale-improved (MESSi; (Díaz-Morales et al. 2017; Randler et al. 2016)) are recommended. Quartile values were used for CCQ classification (upper and lower scores) in this study, in line with prior research (Dosseville et al. 2013; Laborde et al. 2018). Future studies, however, should also aim to develop standard CCQ cutoff scores. Lastly, the period of data collection was between 2021 and 2023 and so the first wave of responses occurred during the COVID-19 pandemic. As a result, UK employees were required to work from home and typical activities, social schedules, sleep preferences, and daily light exposure were subsequently altered. Social restrictions and lockdowns during the pandemic, for example, were shown to alter chronotype preferences and sleep timing habits through changes in circadian rhythm entrainment (Korman et al. 2020; Leone et al. 2020; Rome et al. 2021).

## Conclusions

The English version of the CCQ is a valid and robust tool to capture chronotype estimates in a sample of UK adults. Although the factor structure of the original CCQ was not confirmed, a 2-factor structure (the reduced two-factor model; R2) was still the most parsimonious with superior fit, internal consistency, and convergent validity. Thus, a psychometrically validated two-factor structure of the CCQ was confirmed with distinct, independent amplitude and morningness-eveningness dimensions. Associations with objective sleep parameters further strengthen the reliability of the CCQ. Amplitude (diurnal fluctuation) also demonstrated additional incremental validity in predicting depressive symptoms. Shift workers were shown to have significantly lower amplitude (distinctiveness CCQ-DI scores) which may indicate greater flexibility in tolerating circadian misalignment compared to day workers.

## Supplementary Material

Supplemental Material

## Data Availability

The data that support the findings of this study are available from the corresponding author Teresa C. D’Oliveira (teresa.doliveira@canterbury.ac.uk), upon reasonable request.

## Note

1. This work contains preliminary material revised and reworked from unpublished master’s degree dissertations [author Daniel Lai Jie].
