# Supplementary material for: Validation of the Caen Chronotype Questionnaire: Exploring the added value of amplitude and correlations with actigraphy
Source: Chronobiol Int. 2025 Mar 5;42(3):378–91. doi: 10.1080/07420528.2025.2471887 (PMC11974917; doi:10.1080/07420528.2025.2471887)
Supplement: Supplemental Material [file ICBI_A_2471887_SM4094.pdf]

Supplementary Material

Validation of the Caen Chronotype Questionnaire: exploring the added value of amplitude and correlations with actigraphy

Robert Hickman, Daniel Lai Jie, Sukhi Shergill, Sylvain Laborde, Teresa C. D'Oliveira

Table S1. The Caen Chronotype Questionnaire (CCQ) English translation version
Figure S1. One-factor CCQ model
Figure S2. Two-factor CCQ model
Figure S3. Reduced two-factor model (with AMOS MI recommendations)
Figure S4. AMOS recommended modification indices (MI)

Table S1. The Caen Chronotype Questionnaire (CCQ) English translation version

	**CCQ** Please rate the statements below from 1 (*Totally disagree*) to 5 (*Totally agree*)	
**Item**		**Dimension**
Item 1 (R)	I can work efficiently at any time of the day (R)	CCQ-DI
Item 2 (R)	I feel I can think the best in the morning (R)	CCQ-ME
Item 3	There are moments during the day where I would prefer to avoid any work	CCQ-DI
Item 4	I feel drowsy for a long time after awakening	CCQ-ME
Item 5	If I were to study anything by myself, I would rather do it in the evening	CCQ-ME
Item 6 (R)	My mood stays the same throughout the day (R)	CCQ-DI
Item 7 (R)	I like to get up earlier than necessary, e.g. in order to prepare things for the whole day (R)	CCQ-ME
Item 8 (R)	I can focus at any time of the day (R)	CCQ-DI
Item 9	My work goes better in the afternoon than before noon	CCQ-ME
Item 10 (R)	At any time of the day, my motivation is the same (R)	CCQ-DI
Item 11 (R)	I am usually in an excellent mood in the morning (R)	CCQ-ME
Item 12 (R)	If I need it, I'm able to concentrate at any time of the day (R)	CCQ-DI
Item 13 (R)	I am in my best form in the morning, in the course of the day my energy runs out (R)	CCQ-ME
Item 14	There are moments during the day where I feel unable to do anything	CCQ-DI
Item 15	There are moments during the day when it is harder for me to think	CCQ-DI
Item 16	I feel sluggish in the morning and I warm up slowly during the day	CCQ-ME

Note. **CCQ-DI** = CCQ distinctness amplitude dimension; **CCQ-ME** = CCQ morningness-eveningness dimension; **(R)** = reverse coded items

Figure S1. One-factor CCQ model

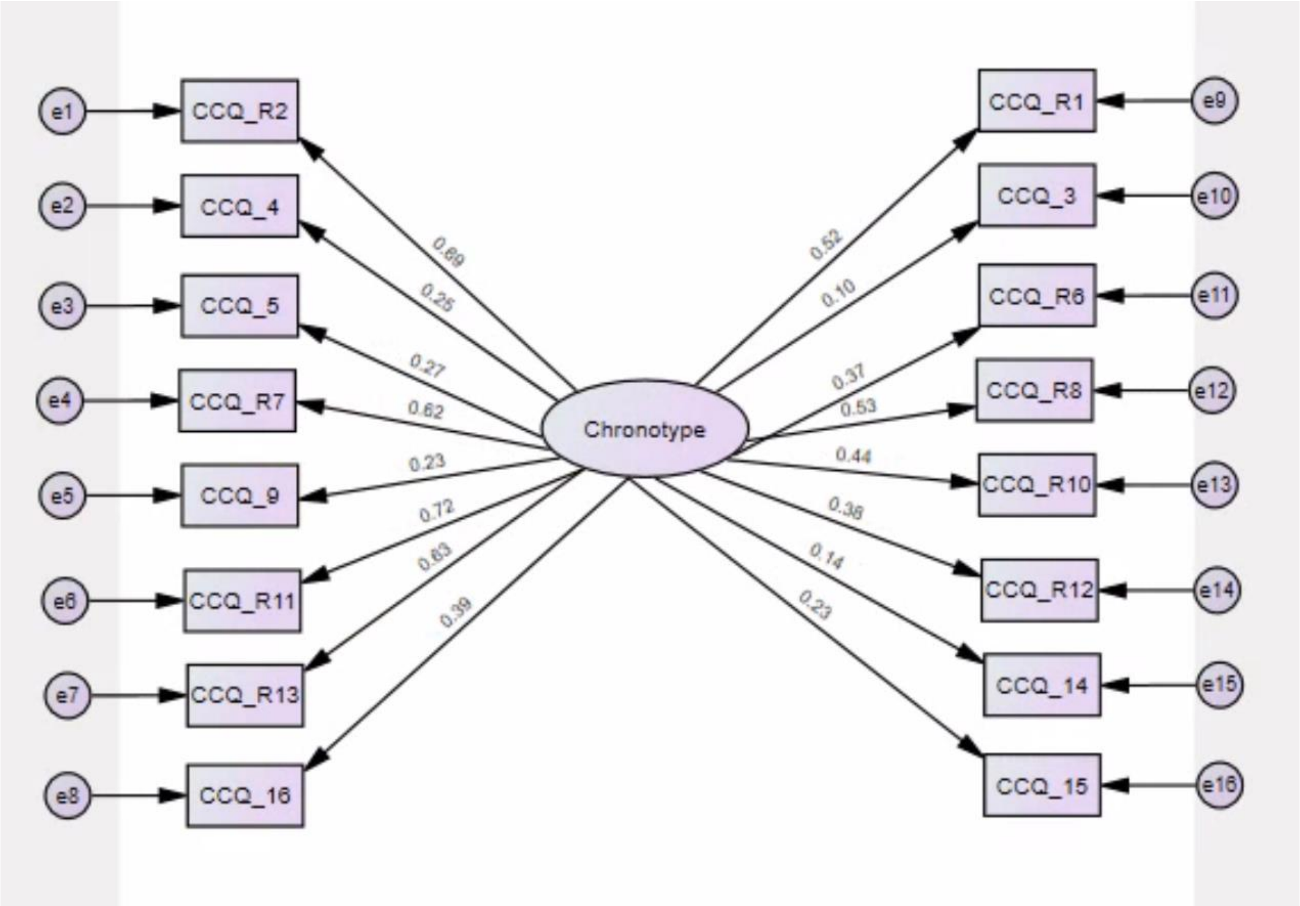

Figure S2. Two-factor CCQ model

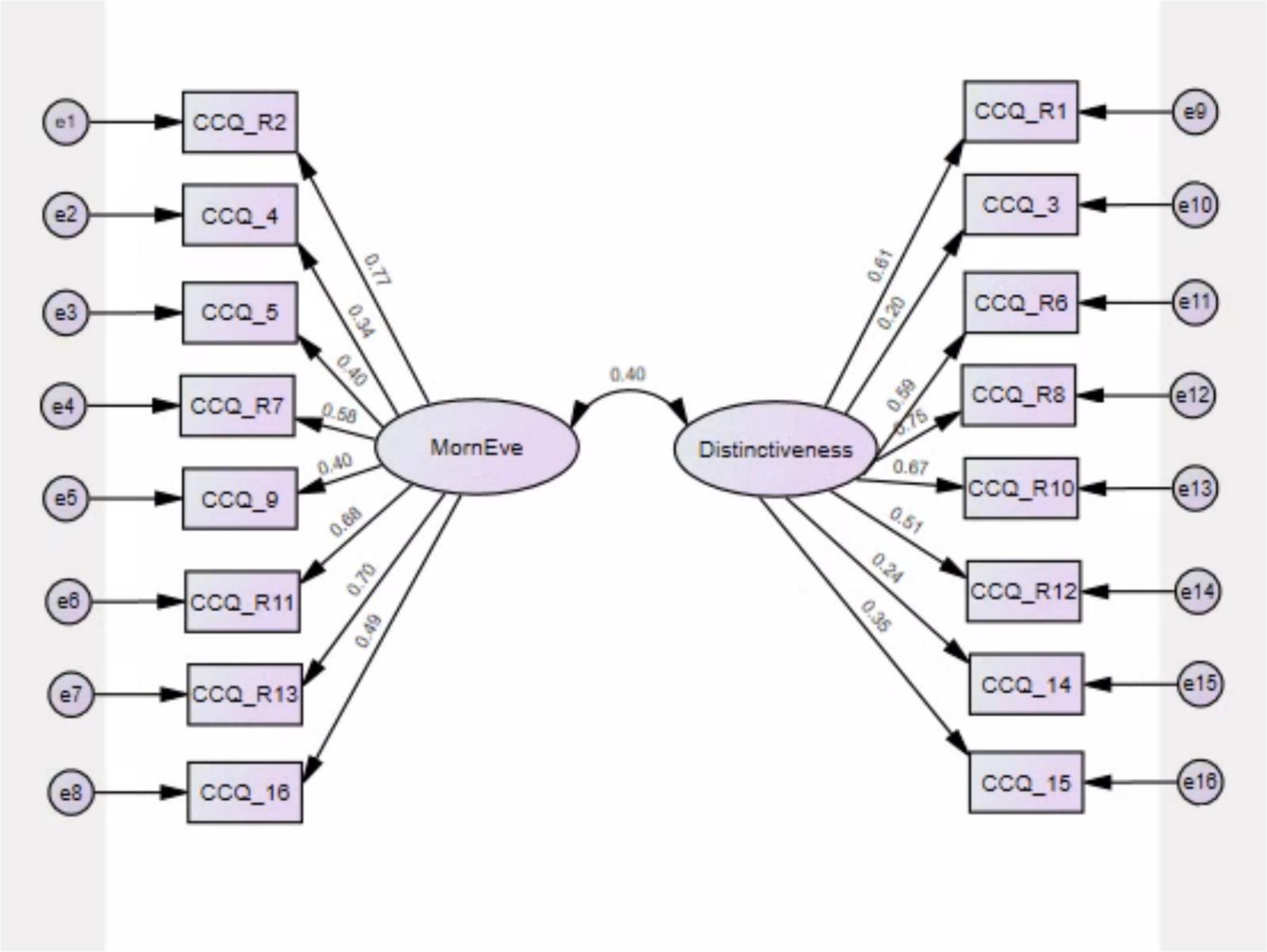

Figure S3. Reduced two-factor model (with AMOS MI recommendations)

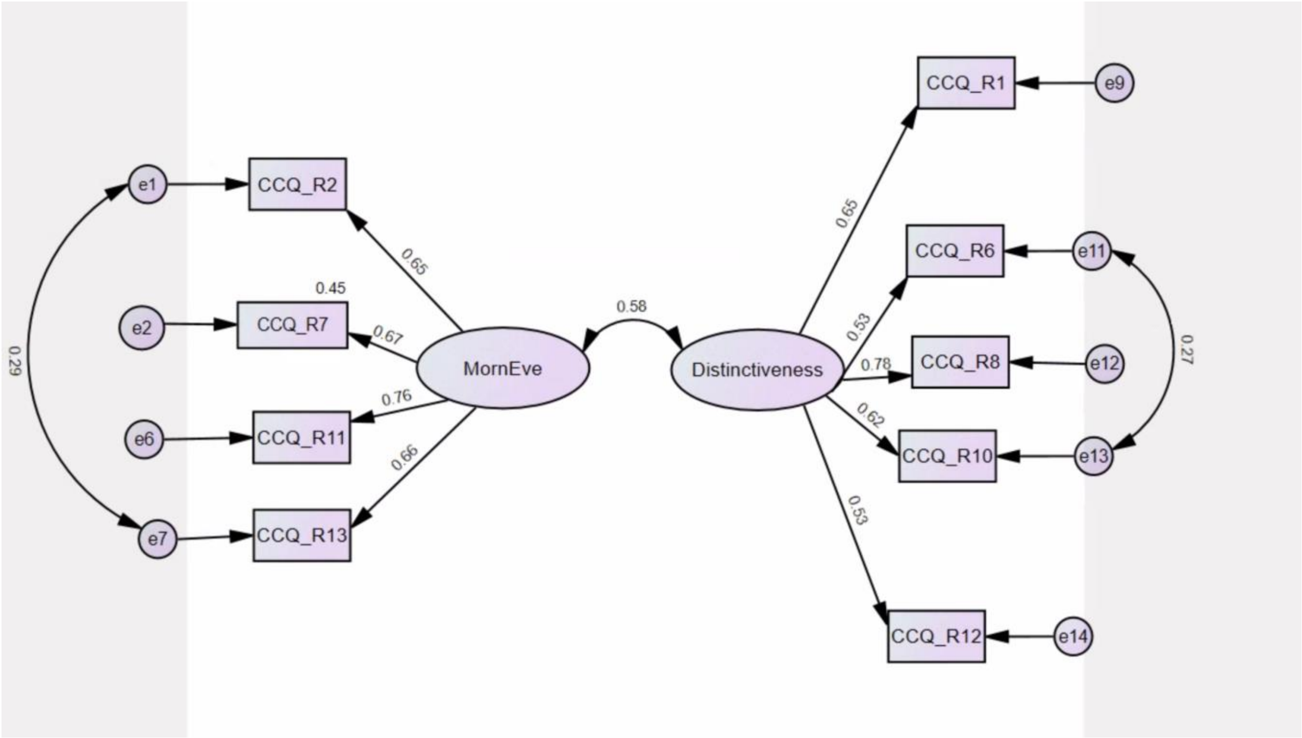

Figure S4. AMOS recommended modification indices (MI)

			**M.I.**	**Par Change**
e2	<-->	Distinctiveness	9.534	.095
e13	<-->	e2	5.779	.098
e11	<-->	e13	29.475	.190
e9	<-->	MornEve	8.740	.096
e9	<-->	e2	8.615	.118
e9	<-->	e13	5.951	-.079
e7	<-->	MornEve	5.242	.073
e7	<-->	Distinctiveness	20.359	-.115
e7	<-->	e11	4.707	-.078
e7	<-->	e9	4.775	-.073
e6	<-->	Distinctiveness	9.719	.081
e1	<-->	e13	10.072	-.110
e1	<-->	e7	14.546	.129
